# Selective Fatty Acid Retention and Turnover in the Freshwater Amphipod *Pallaseopsis quadrispinosa*

**DOI:** 10.3390/biom11030478

**Published:** 2021-03-23

**Authors:** Sami J. Taipale, Erwin Kers, Elina Peltomaa, John Loehr, Martin J. Kainz

**Affiliations:** 1 Department of Biological and Environmental Science, University of Jyväskylä, P.O. Box 35 (YA), 40014 Jyväskylä, Finland; 2 Lammi Biological Station, University of Helsinki, Pääjärventie 320, 16900 Lammi, Finland; john.loehr@helsinki.fi; 3 Institute of Atmospheric and Earth System Research (INAR)/Forest Sciences, University of Helsinki, 00014 Helsinki, Finland; elina.peltomaa@helsinki.fi; 4 Helsinki Institute of Sustainability Science (HELSUS), University of Helsinki, 00014 Helsinki, Finland; 5 WasserCluster Lunz—Biologische Station, Dr. Carl Kupelwieser Promenade 5, A-3293 Lunz am See, Austria; martin.kainz@donau-uni.ac.at

**Keywords:** polyunsaturated fatty acids, nutritional ecology, freshwater, amphipod

## Abstract

Gammarid amphipods are a crucial link connecting primary producers with secondary consumers, but little is known about their nutritional ecology. Here we asked how starvation and subsequent feeding on different nutritional quality algae influences fatty acid retention, compound-specific isotopic carbon fractionation, and biosynthesis of ω-3 and ω-6 polyunsaturated fatty acids (PUFA) in the relict gammarid amphipod *Pallaseopsis quadrispinosa*. The fatty acid profiles of *P. quadrispinosa* closely matched with those of the dietary green algae after only seven days of refeeding, whereas fatty acid patterns of *P. quadrispinosa* were less consistent with those of the diatom diet. This was mainly due to *P. quadrispinosa* suffering energy limitation in the diatom treatment which initiated the metabolization of 16:1ω7 and partly 18:1ω9 for energy, but retained high levels of eicosapentaenoic acid (EPA) and docosahexaenoic acid (DHA) similar to those found in wild-caught organisms. Moreover, α-linolenic *acid* (ALA) from green algae was mainly stored and not allocated to membranes at high levels nor biosynthesized to EPA. The arachidonic acid (ARA) content in membrane was much lower than EPA and *P. quadrispinosa* was able to biosynthesize long-chain ω-6 PUFA from linoleic acid (LA). Our experiment revealed that diet quality has a great impact on fatty acid biosynthesis, retention and turnover in this consumer.

## 1. Introduction

Amphipods are important members of pelagic food webs that connect basal producers and herbivorous zooplankton to upper trophic levels [1,2]. Amphipods also have a crucial role in coupling benthic and pelagic foods and recycling dietary energy in aquatic ecosystems [3,4]. Moreover, they are important food sources for fish and birds [5] and thus passively convey dietary energy across ecosystems. In northern Europe and North America, one of the most common amphipods is the cryophilic *Pallaseopsis quadrispinosa* that dispersed into its present range at the end of the last glaciation [6]. This amphipod has an annual-biennial life cycle and its diet includes phytoplankton, zooplankton and chironomid larvae [7]. *P. quadrispinosa* is usually an epibenthic species that occasionally moves up in the water column inhabiting the profundal, littoral and sublittoral zones [8]. Benthic invertebrates, especially in deep lakes, are often exposed to considerable changes in food availability. Since lipids contain twice as much energy as carbohydrates and proteins [9], aquatic consumers usually store lipids to survive periods of food shortage [10]. More specifically, triacylglycerols and wax-esters are the main classes of storage lipids in copepods, cladocerans and amphipods. However, ways of energy storage vary among species [2,11,12,13]. Previous studies with amphipods have shown high accumulation of neutral lipid fatty acids (NLFA) in polar regions and in habitats where food availability is limited [2], whereas low accumulation of storage lipids was found from amphipods with high food availability [14,15].

In addition to energy reserves, aquatic consumers require phospholipids and cholesterol as membrane lipids [12,16]. Due to the physiological importance of phospholipids their content is usually more stable, but may temporarily decrease, e.g., during embryonic development [11]. Omega-3 and ω-6 long-chain (C_20_–C_22_) polyunsaturated fatty acids (PUFA) are physiologically the most important fatty acids (FA) for aquatic consumers and they are required for maintaining cell membrane fluidity among other functions [17]. Moreover, arachidonic acid (ARA, 20:4ω6), eicosapentaenoic acid (EPA, 20:5ω3) and docosahexaenoic acid (DHA, 22:6ω3) have an important and direct impact on the survival of zooplankton and juvenile fish [18,19] and are a precursor of eicosanoids [20]. Due to the importance of these ω-3 long chain PUFA, their content accumulates in aquatic food webs [21,22]. It is reported that DHA appears to be the most retained FA in copepods and many fish, whereas it is EPA for *Daphnia* and some benthic invertebrates [23,24,25]. EPA is the predominant ω-3 PUFA of amphipods [1,15,26,27,28,29], but they can also contain some DHA. The dietary availability of EPA and DHA also supports the survival of insectivore chicks [30], and their dietary transfer from aquatic to terrestial food webs is critical for birds and mammals since terrestrial plants contain very low amounts of EPA or DHA [31,32,33,34].

Linoleic acid (LA; 18:2ω6) is a precursor for ARA and alpha-linolenic acid (ALA, 18:3ω3) for EPA and DHA [23,35], respectively. Among all freshwater phytoplankton taxa, cryptophytes, chrysophytes, diatoms, dinoflagellates, euglenoids and raphidophytes are able to synthesize EPA and DHA [36,37]. However, not all these taxa are a suitable diet for zooplankton or amphipods [38]. Previous studies suggest that diatoms are the main source of high quality diet for benthic amphipods in the Baltic Sea [39], whereas diatoms and dinoflagellates are the main source of EPA and DHA in polar regions [2]. In *Diporeia*, a previously dominant benthic macroinvertebrate in the Great Lakes, seasonal changes in lipid content were observed due to food availability and dietary quality differences [10,40]. There is laboratory evidence that *Diporeia* retained ARA, EPA and DHA at high levels [40]. However, the potential biosyhthesis of long chain PUFA in freshwater amphipods has not been proven by using ^13^C-enrichment and compound specific carbon isotope measurements [41] as previously used for zooplankton and fish [19,42,43,44]. Therefore, direct evidence of biosynthesis of ARA, EPA and DHA in amphipods from their precursors is still lacking as well as the understanding of how amphipods regulate essential fatty acids in storage and membrane lipids. In addition to understanding the origin of individual FA, investigating FA profiles provides better insight into the overall FA turnover time and FA trophic fractionation as well as how much consumers modify their FA in different lipid fractions. We have used this method previously with herbivorous zooplankton [44], which revealed that *Daphnia* changed their FA in six days at a higher ratio when fed a high-quality diet followed by a moderate-quality diet. The understanding of how consumers fractionate FA from diets of different nutritional quality [45] is also important for FA-based diet modelling [46]. 

Here we examined the overall FA turnover, trophic fractionation and biosynthesis in the freshwater amphipod *P. quadrispinosa* at different trophic conditions. We hypothesized that; (a) the overall FA turnover of *P. quadrispinosa* will be faster and the isotopic trophic fractionation of FA (fractionation of dietary FA in amphipod) lower when fed a high-quality diet (*Diatoma tenuis*) compared to a moderate-quality diet (*Acutodesmus* sp.); (b), ARA, EPA and DHA are first allocated in membranes (phospholipid fatty acid, PLFA) whereas other dietary FA are first allocated to storage and then to membrane lipids; and (c) we predicted that *P. quadrispinosa* is not able to biosynthesize EPA or DHA from ω-3 short-chain PUFA (ALA and stearidonic acid, SDA, 18:4ω3), but it would be able biosynthesize ARA from LA at physiologically adequate levels.

## 2. Materials and Methods

### 2.1. Amphipod Sampling

*P. quadrispinosa* was sampled using plankton dip nets from the Kellolanlähde Spring (Latitude: 61.009188|Longitude: 25.198°) in Hämeenkoski, Finland. The spring is surrounded by stands of *Picea abies* and is oligotrophic with a maximum depth of 3–4 m. The amphipods are sampled by sweeping a plankton net through the submerged vegetation (*Myriophyllum alterniflorum*) that is present on the south end of the pond. About 60 juveniles were selected (2–5 mm body size), transported within 20 min in a bucket filled with spring water to the laboratory where they were placed in clean treated (removing possible chlorine) tap water at 8 °C (tap and spring water were both below 200 µS/cm). *P. quadrispinosa* were classified as juveniles when signs of maturity e.g., marsupium, ovary or testes were absent, using a Leica S4E microscope at 10× magnification. After the amphipods were classified as juveniles, they were placed back into the bucket in a climate room at 8 °C. Six individuals of wild-caught *P. quadrispinosa* were used for lipid analysis.

### 2.2. Phytoplankton Culturing

Phytoplankton cultures of *Acutodesmus* sp. (University of Basel, Switzerland) and *Diatoma tenuis* (CPCC62) were grown in tissue flasks (650 mL) at 18 °C under a 12:12 L-D cycle in MWC (Scandinavian Culture Collection for Algae & Protozoa: MWC/MWC+Se (sccap.dk)) growth medium [47]. When algae cultures reached the stationary phase (dark colour), the cultures were split in half and new MWC medium was added. For ^13^C-labeling algal diets, 5% of the NaHCO_3_ in the MWC growth media was replaced with ^13^C-enriched (99%) NaHCO_3_ when culturing labeled *Acutodesmus* sp. and *Diatoma tenuis*. This treatment was repeated once a week throughout the experiment.

### 2.3. Experimental Setup

Sixty juveniles of *P. quadrispinosa* were placed in eight buckets (seven to eight juveniles per bucket) with 8 L of treated tap water. Juveniles were placed in a climate chamber at 8 °C on a 12 h light-dark cycle. The amphipods were starved for 12 d before the experiment to drain their lipid reserves. The disappearance of lipid droplets was confirmed with stereomicroscopy (Leica DM750). Starvation was done to homogenize lipid reserves and FA profile of collected amphipods. It also enabled the evaluation of how they regulated their FA composition in NLFA and PLFA during starvation and feeding period. For the experiment, one *P. quadrispinosa* was placed in each container (1 L; 28.3 × 22 × 5.5 cm) and kept at 8 °C on a 12 h light–dark cycle. Four individuals (n = 4) were sampled in each experiment every week (on day 7, 14, 21, and 28) resulting in 16 individuals per algal treatment. Each *P. quadrispinosa* were fed initially by 1.5 mg C L^−1^ until day 3 by 3.0 mg C L^−1^of the ^13^C-labelled algae (*D. tenuis* or *Acutodesmus* sp.) resulting in a concentration of 0.75 and 1.5 mg C for each amphipod per day which exceeds food concentrations in previous experiments [40,48]. For lipid analysis whole animals were immediately frozen (−80 °C) and subsequently freeze-dried. Algae were collected at day 28 and filtered (GF/C filter, Whatman International Ltd., Maidstone, UK) to remove water, frozen (−80 °C) and also freeze-dried prior to analysis.

### 2.4. Lipid and Fatty Acid Analysis

Total lipids were extracted from homogenized *P. quadrispinosa* (average weight of one individual was 1.83 ± 0.94 mg) using Folch’s [49] extraction procedure (chloroform:methanol:water mixture of 8:4:3). Internal standard of 1,2-dipentadecanoyl-sn-glycero-3-phosphatidycholine was used to calculate lipid recovery for phospholipid fatty acids [50]. Lipids were fractionated using Bond Elut LRC-SI cartridges (500 mg). The resin of the cartridge was activated using the mixture of chloroform and methanol (1:1). Subsequently, total lipids were dissolved to the 300 µL of chloroform and applied to the cartridge. Chloroform, acetone, and methanol (each 10 mL) were consecutively used to elute neutral lipids, glycolipids, and phospholipids, respectively. Prior sample analysis, i.e., the ability of the silica solid phase extraction (SPE) cartridge to fractionate NLFAs and PLFAs into correct fractions, was tested by spiking glyceryl tripalmitate (Sigma-Aldrich, Saint Louis, Missouri) and 1,2-dipentadecanoyl-sn-glycero-3-phosphatidychloline (Larodan) to SPE-column. The recovery percentage for the NLFA and PLFA standard was 68 ± 8% and 93 ± 7%, respectively. Approximately 11% and 5% of the NLFA standard ended up in the glycolipid (GL) or phospholipid (PL) fraction, whereas the PLFA standard was not detected in NL or GL fractions. 

Fatty acids of NLFA and PLFA class were methylated using acidic conditions (1% sulphuric acid in methanol) to fatty acid methyl esters (FAME). FAME were analyzed with a gas chromatograph (Shimadzu Ultra, Kyoto, Japan) equipped with mass detector (GC-MS) and using helium as a carrier gas (linear velocity = 36.3 cm s^−1^). Two of four replicates were run using a Phenomenex^®^ (Torrance, CA, USA) ZB-FAME column (30 m × 0.25 mm × 0.20 µm) and the other two replicates were run with a DB-23 column (60 m × 0.25 mm × 0.25 µm) with 5 m guard column in each. The temperature of the injector was 270 °C and we used splitless injection mode (for 1 min). The temperatures of the interface and ion source were 250 °C and 220 °C, respectively. The following temperature program was used with ZB-FAME column: 50 °C was maintained for 1 min, then the temperature was increased at 10 °C min^−1^ to 130 °C, followed by 7 °C min^−1^ to 180 °C, and 2 °C min^−1^ to 200 °C and held for 3 min, and finally heated at 10 °C min^−1^ to 260 °C. Total program time was 35.14 min and solvent cut time 9 min. The temperature program for the DB-23 was the following: 60 °C was maintained for 1 min, then increased at 30 °C min^−1^ to 130 °C, followed by 7 °C min^−1^ to 180 °C, and 1.5 °C min^−1^ to 220 °C and held for 5 min, and finally heated at 30 °C min^−1^ to 240 °C and held for 3 min. Fatty acids were identified by the retention times (RT) and using specific ions [37], which were also used for quantification. FAME concentrations were calculated using calibration curves based on known standard solutions (15 ng, 50 ng, 100 ng and 250 ng) of a FAME standard mixture (GLC standard mixture 566c, Nu-Chek Prep, Elysian, MN, USA) and using recovery percentage of internal standard for PLFA (> 72%). The Pearson correlation coefficient was >0.99 for each individual fatty acid calibration curve. 

### 2.5. Gas Chromatography Combustion Stable Isotope Ratio Mass Spectrometry (GC-C-IRMS)

The δ^13^C values of FAME were determined using a GC-C TA III connected to an isotope ratio mass spectrometer (IRMS, DELTAPLUSXP, Thermo Co., Bremen, Germany) at the Interuniversity Centre for Aquatic Ecosystems Research WasserCluster Lunz (Lunz am See, Austria). Fatty acids were separated using a 60 m DB-23 column (0.25 mm × 0.15 mm) and then oxidized to carbon dioxide in an oxidation reactor at a temperature of 940 °C with the reduction reactor kept at 630 °C. The temperature program of the GC column started at 60 °C and was kept for 1 min at 60 °C, after which the temperature was raised by 30 °C min^−1^ to 175 °C, and then by 2.6 °C min^−1^ to 245 °C, and held at that temperature for 17 min. The total run time was 48.03 mi. The injector temperature was kept at 270 °C. The samples were run against an internal standard, 1,2-Dinonadecanoyl-sn-Glycero-3-Phosphatidylcholine (Larodan, δ^13^C = −28.43‰), which was used for drift and linear correction. The calculated precision for standard FAME was ± 0.4‰ and the accuracy was ± 0.3‰. The δ^13^C value of individual FAME was manually calculated using individual background values. The δ^13^C value of FA was calculated from the δ^13^C value of FAME by correcting the methyl group [51]. 

### 2.6. Statistics

Comparison between experiments was made using ANOVA and Tukey’s post hoc test, in cases of unequal variances we used Welch ANOVA and Dunnets T3 test. The limit of statistical significance in all tests was set at *p* < 0.05. ANOVA and Welch ANOVA were conducted using IBM SPSS (version 24.0; IBM 2016) software. Bray Curtis similarity matrix of FA contributions was created using Primer 7 [52] and nonmetric multidimensional scaling (nMDS) analysis [53] was applied. Hierarchical cluster analysis were used to create 80% similarity clusters on nMDS plot. Pearson’s correlation (r > 0.6) of nMDS axis and FA was used to show main overlay vectors. Permutational multivariate analysis of variance (PERMANOVA) [54] was used to test whether these differences in the FA composition were statistically significant among wild (before the start of the feeding experiment), starved (day 0) and in green algae and diatom treatments. Treatment and day (7, 14, 21, 28) or lipid fractions (NLFA, PLFA) were used as factors in two factors PERMANOVA (Primer 7). Additionally, one factor (treatment) PERMANOVA was run for testing differences in the PLFA and NLFA fraction. PERMANOVA was run with unrestricted permutation of raw data and type III sums of squares. Similarity percentages (SIMPER) were used to identify similarities within treatment and lipid fractions. Additionally, SIMPER was used to identify dissimilarities in FA composition between treatments and lipid classes (Table 2).

We calculated the proportion of *P. quadrispinosa* FA in the food treatments that derived from the original (wild + starved) and new food sources (*Acutodesmus* sp. or *Diatoma tenuis*). This was made by comparing the actual *P. quadrispinosa* FA profiles to hypothetical *P. quadrispinosa* FA profiles which was obtained by “mixing” the initial (starved) *P. quadrispinosa* FA profiles and algal diet to assess the proportion of the initial diet that maximized the fit between the observed and predicted profiles. Specifically, we used the following algorithm:

Predicted diet FA profile = X × (initial FA profile) + (1−X) × (subsequent FA profile),

where X equals the hypothetical proportion of the original dietary FA retained by *P. quadrispinosa*. We then used the Solver^®^ function in Excel^®^ to find the value of X that maximized the correlation coefficient (r) between the observed and predicted FA profiles.

We used a two-source carbon isotope mixing model, IsoError software, version 1.04 [55], to assess how much of FA originated from the diet and to evaluate how much of ARA, EPA, DHA in amphipods originated from dietary ALA or LA. The mean proportion of assimilated diet (*f_A_* = green algae or diatom) by P. quadrispinosa was calculated using the following mixing model:

(*f_A_*) = δ_M_ − δ_B_/δ_A_ − δ_B_,

where δ*_M_* represent δ^13^C values of *P. quadrispinosa* (day 7, 14, 21 or 28), δ*_A_*represent δ^13^C values of algal diet (green algae or diatom), and δ*_B_*represent δ^13^C value of starved amphipod (day 0). Isotopic fractionation was not taken into account since the variation of δ^13^C values of amphipods and diets varied more than previously measured isotopic fractionation (e.g., −4‰; [51]) due to the ^13^C-enrichment (Supplemental Table S1).

Additionally, we calculated the content of EPA and ARA in *P. quadrispinosa* that derived from the diet. This was calculated by multiplying original EPA and ARA content of *P. quadrispinosa* by the contribution from the diet as suggested by the IsoError model (Table 3).

## 3. Results

### 3.1. Total Lipid, NLFA and PLFA Content of Wild, Starved and Re-fed Amphipods

**Figure 1:**
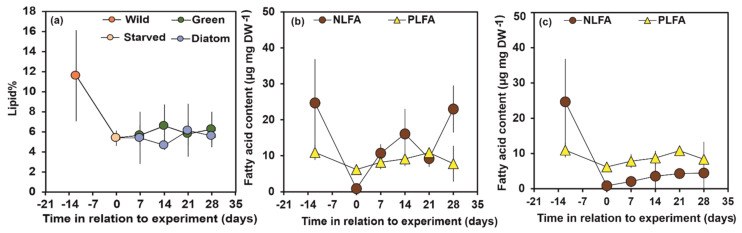
(**a**) Lipid content of *Pallaseopsis quadrispinosa* in wild (day −12), starved (day 0) and in green algae (*Acutodesmus* sp.) and diatom (*Diatoma tenuis*) experiments. Day refers to the age of initial wild-caught amphipods (−12) in relation to the start (day 0, starved 12 days) of the feeding experiment. Total NLFA and PLFA content (**b**) in green algae (*Acutodesmus* sp.) and (**c**) diatom (*Diatoma tenuis*) experiments.

The relative lipid content was 12 ± 5% (mean ± std.) of dry weight (DW)in wild-caught P. quadrispinosa from Kellolanlähde Spring (Figure 1a). Lipids of *P. quadrispinosa* did not differ statistically between treatments (ANOVA: F1, 7 = 2.89, *p* = 0.14). The total NLFA and PLFA content of the wild juvenile amphipods *P. quadrispinosa* were 25 ± 12 and 11 ± 2 µg FA mg DW^−1^, respectively. After 12 days of starvation, the total content of NLFA was only 3 ± 1% of initial content, whereas the PLFA remained higher (57 ± 3% of initial, Figure 1b). During the whole experiment the total NLFA content of *P. quadrispinosa* with green algae diet exceeded the PLFA (Figure 1b) content whereas PLFA exceeded NLFA in the diatom experiment (Figure 1c). The NLFA content of *P. quadrispinosa* was higher (ANOVA: F_1, 25_ = 12.4, *p* = 0.002) in the green algae than in the diatom experiment, whereas PLFA of *P. quadrispinosa* did not differ between treatments (ANOVA: F_1, 28_ = 0.38, *p* = 0.54).

### 3.2. FA Profiles of Wild, Starved and Re-Fed Amphipods

**Figure 2:**
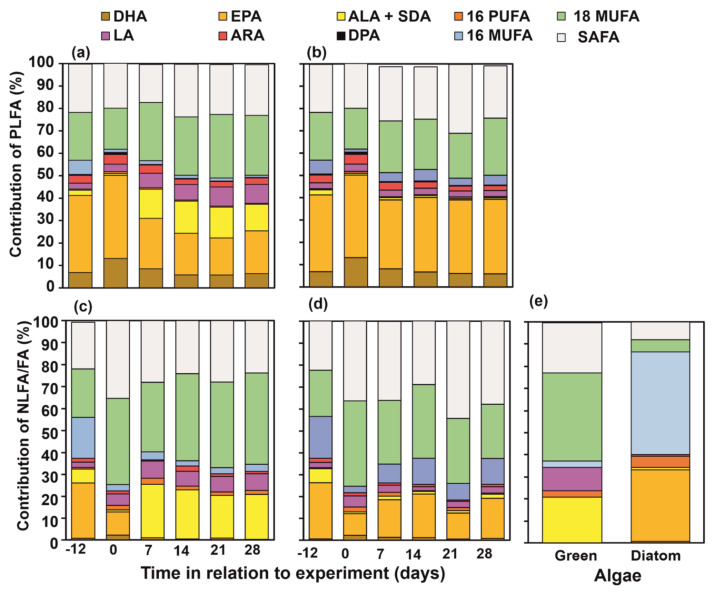
Phospholipid fatty acid (PLFA) and neutral lipid fatty acid (NLFA) and composition of *Pallaseopsis quadrispinosa* in (**a**,**c**) green algae (*Acutodesmus* sp.) and (**b**,**d**) diatom (*Diatoma tenuis*) treatments. Day refers to the age of initial wild-caught amphipods (−12) in relation to the start (day 0, starved 12 days) of the feeding experiment. (**d**) Diet fatty acid composition: green (*Acutodesmus* sp.) and diatom (*Diatoma tenuis*). (**e**) Total fatty acid composition of green algae (*Acutodesmus* sp.) and diatom (*Diatoma tenuis*) used in the experiment. Abbreviations: SAFA = saturated fatty acids, 18 MUFA = 18 monounsaturated fatty acids, 16 MUFA = 16 monounsaturated fatty acids and 16 PUFA = 16 polyunsaturated fatty acids.

FA profiles of PLFA and NLFA varied from each other (Supplemental Tables S1 and S2). Similarity of FA profiles of *P. quadrispinosa* of wild, starved and re-fed individuals was higher in the PLFA than in the NLFA fraction (Table 1). When taking lipid fraction and treatment into account in two factor PERMANOVA, treatment explained 55% (t = 73.6, P(perm) = 0.001) and lipid fraction 18% (t = 72.0, P(perm) = 0.001) of the variation of amphipod NLFA and PLFA profiles. NLFA (PERMANOVA: t = 3.1–10.1, P(perm) = 0.001–0.009) and PLFA (PERMANOVA: t = 1.9–7.7, P(perm) = 0.001–0.022) profiles of amphipods differed among treatments. EPA, 16:1ω7, 18:1ω9 and 16:0 were the most abundant NLFA in wild-caught *P. quadrispinosa*, whereas EPA, 18:1ω9 and 16:0 were the most abundant PLFA in *P. quadrispinosa* (Figure 2). The 16:1ω7 was more abundant in NLFA (18 ± 6% of all FA) than in PLFA (6 ± 2% of all FA) of wild-caught *P. quadrispinosa*, whereas DHA was more abundant (7 ± 2% of all FA) in PLFA than in NLFA (0.8 ± 0.4% of all FA).

**Figure 3:**
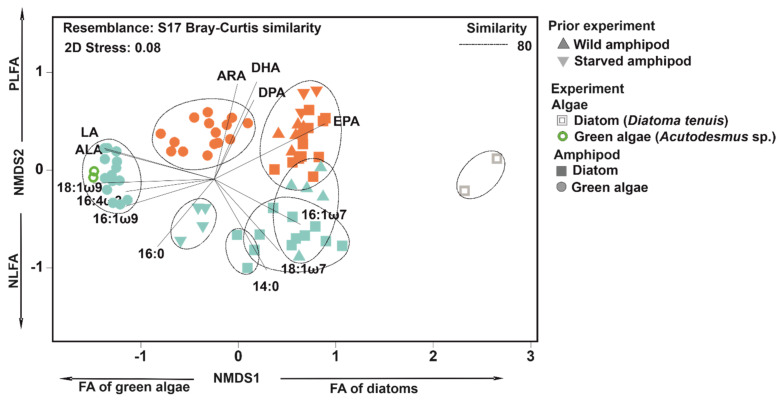
Nonmetric multidimensional scaling (NMDS) for fatty acid profiles of total lipids neutral lipids (NL, cyan colour of symbols) and phospholipids (PL, orange colour of symbols) of amphipods (*Pallaseopsis quadrispinosa*) in green algae and diatom treatments including wild (day −12) and starved individuals (day 0). Shape of symbols refers to the treatments and colour of symbols to lipid fraction. Vectors show Pearson’s correlations (r > 0.8) of individual fatty acids with the axis of NMDS1 and 2. Dashed-lined clusters are based on >80% similarity in Cluster analysis.

After 12-days starvation, 18:1ω9 and 16:0 formed together 50% of all NLFA, and the contribution of EPA was only 11 ± 3% of all NLFA. PLFA profile of starved amphipods was similar in wild-caught individuals (Figure 3), but the contribution of DHA was higher in starved individuals than in wild-caught juveniles. The nMDS analysis separated *P. quadrispinosa* feeding on green algae or diatom (Figure 3). Feeding on green algae diet increased the contribution of LA and ALA in PLFA and NLFA. Correspondingly, feeding on diatom increased the contribution of 16:1ω7 and EPA in the NLFA fraction of amphipods, but not in the PLFA fraction (Figure 2 and Figure 3). Moreover, the nMDS closely clustered green algae (*Acutodesmus* sp.) and NLFA of amphipods fed on green algae, whereas NLFA of amphipods fed on the diatom (*D. tenuis*) were loosely related with the FA profile of diatom (Figure 2). The PLFA profile of amphipods in the green algae experiments had higher dissimilarity with the diet FA profile than the NLFA fraction of amphipods, whereas amphipods in the diatom experiment were the opposite (Table 2).

### 3.3. NLFA and PLFA Composition of Wild, Starved and Re-Fed Amphipods

**Figure 4:**
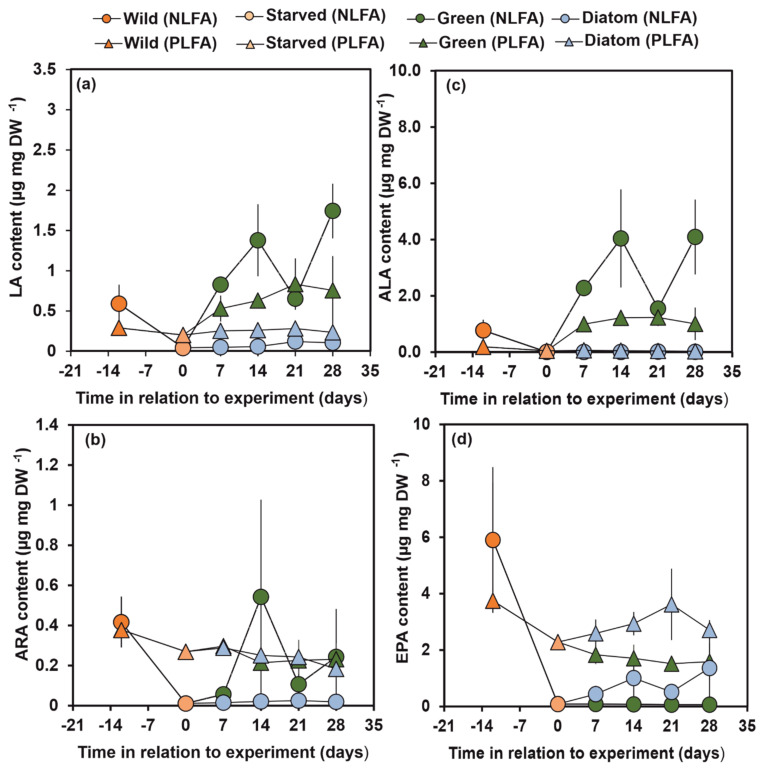
(**a**) LA, (**b**) ALA, (**c**) ARA, and (**d**) EPA content of *Pallaseopsis quadrispinosa* in PLFA and NLFA fraction in wild (day −12), starved (day 0) and in green algae (*Acutodesmus* sp.) and diatom (*Diatoma tenuis*) experiments. Day cites to the age of initial wild amphipods (−12) in relation to the start (day 0, starved 12 days) of the experiment starved.

The LA content was higher in NLFA and PLFA fraction of amphipods fed on green algae than in wild, starved or diatom-fed amphipods (Welch ANOVA: F_3, 12.3/9.193_ = 27.4/11.39, *p* < 0.0001; Figure 4a). In contrast, wild amphipods had higher ARA in PLFA than in individuals fed on green algae or diatoms (Welch ANOVA: F_3, 12.59_ = 19.8, *p* = 0.001, Figure 4b). Amphipods fed on green algae or diatom did not differ in their ARA (Dunnett T3: *p* = 1.000, Figure 4b). However, high ARA content in NLFA of *P. quadrispinosa* fed on green algae was measured on days 14 and 28. 

The ALA content in NLFA and PLFA was significantly higher in amphipods exposed to green algae than in wild, starved or those given a diatom diet (Welch ANOVA: F_3, 12.082/9.765_ = 24.2/71.3, *p* < 0.001; 4c). While the content of ALA varied in NLFA of amphipods fed on green algae, ALA remained relatively stable in PLFA (Figure 4c). The EPA content in PLFA was higher in wild individuals than in starved, green-algae-fed or diatom-fed individuals (Welch ANOVA: F_3, 9.155_ = 31.4, *p* < 0.001). Furthermore, the EPA content in PLFA was significantly higher in amphipods feeding on diatoms than on green algae (Dunnett T3: *p* < 0.001, Figure 4d).

### 3.4. Overall Fatty Acid Turnover in Different Lipid Fraction of P. quadrispinosa

**Figure 5:**
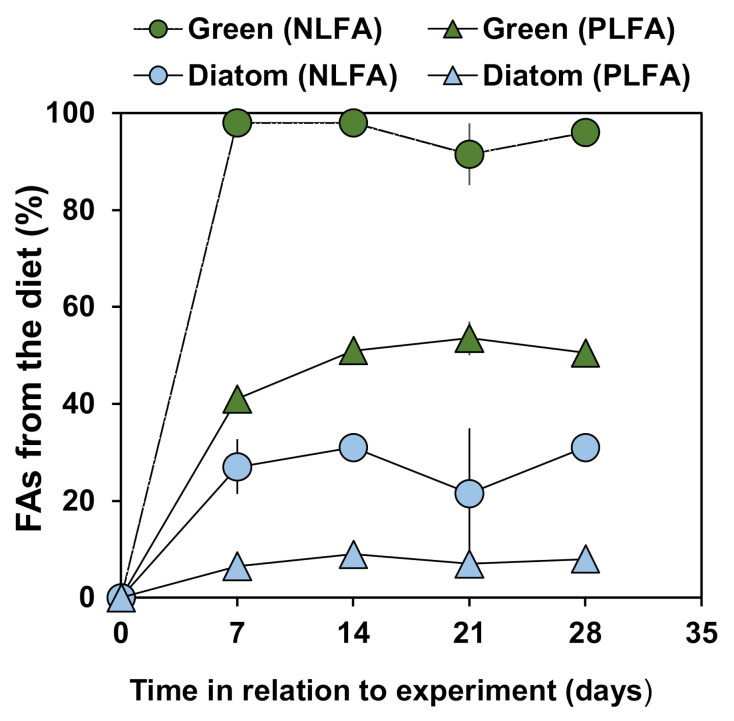
The contribution of fatty acids (FAs) derived from the diet in an amphipod (*Pallaseopsis quadrispinosa*). Two lipid fractions assessed for (a) green algae (*Acutodesmus* sp.) and (b) diatom (*Diatoma tenuis*) diets. The values were obtained from a mixing model that obtained the best fit between the observed FA profile and a hypothetical mixed profile derived using end members from the initial diets (see Methods).

The overall turnover of NLFA and PLFA in *P. quadrispinosa* differed greatly between the algal treatments (Figure 5). The amphipods feeding on green algae renewed their NLFA fully in seven days, whereas *P. quadrispinosa* feeding on diatom renewed only 27 ± 5% of their NLFA at the same time. Correspondingly, 41–51% of amphipod PLFA derived from the green algae, but only 7–9% originated from the diatoms based on linear comparison of full FA profiles. The mixing model fit was very good (R^2^ > 0.95, Error SS < 107) in the green algae treatment including both lipid fractions. The mixing model for the diatom treatments resulted in a relatively good fit for the expected composition in PLFA (R^2^ > 0.87, Error SS < 203), whereas the expected composition had a lower fit in NLFA (R^2^ > 0.63, Error SS < 382). The levels of EPA, DHA and ARA were higher in the amphipod PLFA than in their diet and formed most of the lack of fit in the hypothetical FA profile based on diatom FA Additionally, diatom-fed amphipods had a higher contribution of 18:1ω7 and 16:0 as the model predicted. In the green algae treatment, the contribution of ALA and LA was lower in the amphipod than the model predicted.

### 3.5. Turnover of Individual FA Based on δ^13^C of NLFA and PLFA 

Carbon isotopic values of FA in NLFA and PLFA were more depleted in wild than in starved amphipods (Supplemental Table S3). *D. tenuis* was more enriched in ^13^C than *Acutodesmus* sp. during the experiment resulting in more enriched δ^13^C values of PLFA and NLFA in amphipods of the diatom than green algae treatment. LA and 18-carbon MUFA were the most ^13^C-enriched NLFA and PLFA in amphipods fed on green algae. Additionally, saturated fatty acids were more enriched in NLFA than in PLFA. In the diatom treatment, 14:0 and 16:1ω7 were the most ^13^C-enriched NLFA and PLFA. Additionally, EPA was highly ^13^C-enriched in NLFA in the diatom treatment, whereas EPA together with other long-chain PUFA became increasingly more ^13^C-enriched in PLFA. 

**Figure 6:**
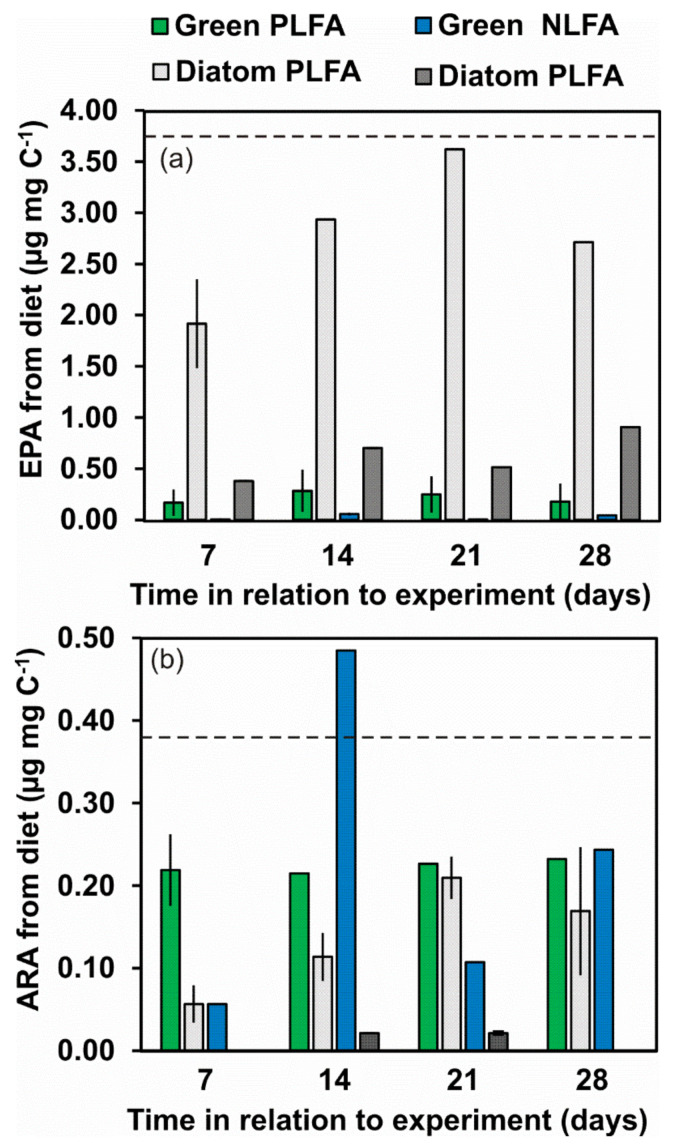
Calculated amount of eicosapentaenoic acid (EPA) (**a**) and arachidonic acid (ARA) (**b**) in NLFA and PLFA fraction which originated from the diet (green algae or diatom). Dashed line shows the EPA and ARA content of wild individuals that can be kept as desirable content of EPA and ARA.

When using the δ^13^C values of FA of algal diets and the δ^13^C values of PLFA and NLFA of starved amphipods in two-source mixing model calculations, the amphipod retained FA selectively in different lipid fractions (Table 3). Already after 7 d, *P. quadrispinosa* had almost fully changed 18:1ω7, 18:1ω9, LA and ALA in both fractions in green algae treatment, whereas 14:0, 16:0 and 18:0 was only partly changed. According to the mixing model calculations, 100% of LA and 74% of ARA in the amphipod originated from green algae already after 7 d in both lipid fraction. After 14 d, all ARA originated from the green algae in both lipid fractions. Only 9–17% and 2–4% of EPA and DHA of the amphipods originated from dietary ALA in PLFA, whereas 5–77% of EPA originated from dietary ALA in NLFA. However, when expressed as mass fractions (Figure 6), only 0.22 ± 0.06 µg EPA mg DW^−1^ and 0.03 ± 0.06 µg EPA mg DW^−1^ in PLFA and NLFA originated from green algae. These values were <6% of the EPA content of membranes of wild amphipods, whereas this amphipod regained 59 ± 2% of ARA from wild amphipod level in the green algae experiment.

The turnover of individual FA in amphipods fed on diatoms was slower in relation to green algae and only PLFA of 14:0 and 16:1ω7 fully originated from the diet; however, FA turnover was higher in NLFA (e.g., >91% for 14:0, 16:1ω7, 18:1ω9,LA, EPA). *P. quadrispinosa* gradually changed their membrane EPA and DHA in the diatom experiment and already after 14 days 100% of EPA was derived from the diatom diet, whereas 54 ± 6% of DHA originated from their diet. A similar trend was also observed for ARA, which *P. quadrispinosa* biosynthesized from dietary LA. Since only traces of ARA and DHA were found in NLFA, it was not possible to calculate their turnover rate in NLFA. In membrane lipids, diatoms contributed 2.80 ± 0.70 µg EPA mg DW^−1^ and 0.14 ± 0.07 µg ARA mg DW^−1^ (Figure 6). These mass fractions accounted for 75 ± 19% and 36 ± 17% of the wild amphipods membrane EPA and ARA content, respectively.

## 4. Discussion

The high content of storage lipids in wild-caught *P. quadrispinosa* in the Kellolanlähde Spring was similar to storage lipids in amphipods from polar regions [2]. This indicates that food availability may be limited in springs and juvenile *P. quadrispinosa* try to store lipids [14,39] for reproduction and survival. EPA was the main FA and ω-3 PUFA in NLFA and PLFA suggesting high availability of EPA in Kellolanlähde Spring for *P. quadrispinosa* at the time of sampling. After 12 days of starvation, all EPA was consumed from storage lipids, whereas EPA remained almost at its initial level in the cell membranes indicating the importance of EPA for optimal cell membrane functioning in *P. quadrispinosa*. In addition to EPA, PLFA included some old DHA (~7% of all FA) retained from prior of the feeding experiment, but DHA contributed only <1% of storage FA, which also suggests a high physiological importance of DHA for optimal performance of membranes in this amphipod. Actually, high EPA and DHA contents are required for membrane fluidity during cold periods especially in the Antarctic marine ecosystem [56] which may explain the high content of EPA and DHA in wild-caught *P. quadrispinosa* from the Kellolanlähde Spring. In a previous study, Kolanowski et al. [57] showed EPA to be more abundant than DHA in different freshwater gammarid species. In contrast, an equal contribution of EPA and DHA has been found in *Diporeia* [40] and *Gammaracanthus lacustris* [58], and a higher contribution of DHA than EPA in endemic gammarids in Lake Baikal [59] Altogether, most of the gammarid amphipods, including *P. quadrispinosa*, seem to have ω-3 long-chain PUFA profiles similar to other crustacean invertebrates [60]. The contribution of EPA in PLFA and NLFA of the wild *P. quadrispinosa* was higher than previously reported in gammarids (5–16% of all FA; [57,58]). The EPA in juvenile *P. quadrispinosa* was also higher than previously observed in zooplankton (8–18% of all FA) in a large boreal lake [22], and the cumulative contribution of EPA and DHA is equivalent to previous findings reported for calanoid zooplankton (26–45% of all FA). However, even though the total FA content of *P. quadrispinosa* and other gammarid amphipods [57] is much higher than in benthic invertebrates [61], the total FA content as well as EPA and DHA is usually highest in freshwater calanoids [62]. The observation that *P. quadrispinosa* has similar EPA and DHA levels to calanoids highlights that *P. quadrispinosa* may have relatively high nutritional value for fish. The similar FA profile of wild *P. quadrispinosa* for those individuals fed on diatoms (*D.a tenuis*) suggests that diatoms may be a key diet component of *P. quadrispinosa*, which could explain the high retention for EPA. In contrast to EPA, we found only a relatively low (<5%) contribution of LA, ALA and SDA in wild *P. quadrispinosa* as previously found with *G. lacustris* [58]. Kolanowski et al. [57] reported a relatively high contribution of LA from different freshwater gammarid species, suggesting relatively high flexibility of FA patterns of freshwater gammarid species, which is most likely the result of different diet compositions.

Similar to *Eudiaptomus* [62], *P. quadrispinosa* maintained a high PLFA content, whereas the NLFA content was almost completely depleted after 12 d of starvation (<2 µg FA mg^−1^ DW^−1^). Moreover, starvation did not decrease EPA in PLFA, whereas the EPA content in NLFA dropped to <10%. The algal refeeding experiment provided important information on the functioning of these consumers after starvation to survive. According to our overall FA turnover model, the amphipods changed more of their FA with the moderate (green alga) than with high-quality diet (diatom), which is contrary to our hypothesis I and previous findings with *Daphnia* [44]. However, this is mainly due to the fact that *P. quadrispinosa* seemed to lack energy since they start to metabolize some FA, which may be because *D. tenuis* has lower carbon and carbohydrate content than *Acutodesmus* sp. due to the silica cell wall [63,64]. Since the nutrional content was three times higher than previously used with diatoms for a benthic freshwater amphipod [40], the demand for a high food content of *P. quadrispinosa* is surprising but indicates a difference in nutritional ecology among freshwater amphipods.

To survive periods of low food, benthic invertebrates frequently store energy in the form of lipids during periods of high food abundance [10]. Therefore, it is possible that selective FA assimilation is related to the fat content of the gammarid. Dietary FA are assimilated without modification when the fat content of *P. quadrispinosa* is high, but after starvation their energy reserves are low and this consumer needs to use some FA for energy. Therefore, it is likely that *P. quadrispinosa* rapidly metabolized assimilated 16:1ω7c from the diatom diet, and thus the contribution of 16:1ω7 of gammarid remained at a low level throughout the experiment. This is also supported by the fact that 16:1ω7c content in storage lipids of *P. quadrispinosa* was high (~5 µg FA mg DW^−1^) in wild-caught juveniles, and also when food deprived, *P. quadrispinosa* catabolized 16:1ω7 almost fully (~0.05 µg FA mg^−1^ DW^−1^). Moreover, the EPA and 16:1ω7 contents dramatically dropped when deprived of food, while the relative content of oleic acid increased many times in the NLFA fraction. Therefore, it is possible that *P. quadrispinosa* used stored EPA for maintaining membrane functions and retained energy from 16:1ω7. In addition to EPA, DHA and 18:1ω7 has prevalence retention from the diatom diet, meanwhile 16:1ω7, 18:1ω9 and LA were not highly retained but rather metabolized. This experiment shows that under food limitation consumers retain physiologically important FAs to optimize performance of cell membranes, and metabolizes remaining FA, whereas dietary FA are not modified when supplied in high quantity.

In line with our second hypothesis, our algal experiment showed selective ω-3 PUFA uptake for membranes indicating that consumers need to replace and/or add new functional carbon to the cell membranes rather than stocking newly acquired carbon to storage lipids. This selective allocation to membrane lipids is important for cell functioning during somatic development, e.g., somatic growth and eventually reproduction. Even though *P. quadrispinosa* feeding on the diatom diet was not able to regain high lipid resources, they were able to increase EPA in their membranes almost to the wild-caught level, whereas EPA in membranes did not increase when *P. quadrispinosa* was feeding on green algae. Moreover, ALA from green algae was mainly stored and not allocated into membranes at high levels. Since ARA content was much lower than EPA in membranes, it seems that physiological demand of membranes for ARA is much lower than for EPA. Moreover, ARA content of membranes in *P. quadrispinosa* remained at the same level throughout the green algae treatment, whereas ARA content of NL varied greatly. Thus, it is difficult to estimate if ARA was preferentially allocated to membranes over neutral lipids or not.

In accordance with our third hypothesis, *P. quadrispinosa* biosynthesized ω-6 long-chain PUFA, but not ω-3 PUFA from their precursors. More specifically, similar to *Daphnia* [65], *P. quadrispinosa* biosynthesized ARA from its ω-6 precursor LA and ARA fully originated from dietary LA after seven days. In contrast, *P. quadrispinosa* did not biosynthesize EPA from ALA efficiently. Even though EPA became more ^13^C-labelled with time in NLFA and PLFA of amphipods fed on green algae, the EPA content decreased from 5 to 2–3 µg mg DW^−1^. Therefore, it appears that *P. quadrispinosa* retained EPA at least at minimum levels, but was not able to increase EPA, which is similar to *Daphnia* [44]. The apparent conversion from LA to ARA, but lacking production of EPA from ALA, suggests that *P. quadrispinosa* may more efficiently retain ω-3 than ω-6 long-chain PUFA. Differences in biosynthesis ability for ARA and EPA from their precursors cannot be the result of lacking energy [32]. Further, the same enzymes are used for the production of ARA than for EPA, but it seems that *P. quadrispinosa* preferred the production of ω-6 PUFA over ω-3 PUFA [66]. Since zooplankton has also very limited ability for EPA / DHA biosynthesis from the dietary ALA [44,62,66], the role of phytoplankton in supplying dietary EPA and DHA seems to be crucial for herbivorous aquatic consumers.

In conclusion, our study demonstrates that the nutritional status of the amphipod influences FA biosynthesis from the diet, and increases our understanding of the importance of ARA, EPA and DHA in the overall functioning of the food chain.

## Figures and Tables

**Table 1:** Similarity percentage (SIMPER) analysis was used to assess similarity between NLFA and PLFA fraction in different treatments. Listed FA contributes >70% of all similarity within lipid class of different days.

Average Sim. (PLFA, NLFA, %)	Main FA (PLFA/NLFA)
Wild (92.1, 85.4)	EPA, 18:1ω9, 16:0, DHA / EPA, 16:1 ω7, 18:1ω9, 16:0
Starved (89.7, 91.3)	EPA, 18:1ω9, DHA, 16:0 / 18:1ω9, 16:0, 18:0, EPA
Green (87.1, 87.3)	18:1ω9, EPA, 16:0, ALA / 18:1ω9, 16:0, EPA
Diatom (86.6, 81.0)	EPA, 16:0, 18:1ω9, 18:1ω7 / 16:0, EPA, 18:1ω9, 18:1ω7

**Table 2:** Average dissimilarities (%) of pairwise comparison of fatty acid profiles of wild, starved (day 0), green algae and diatom-fed amphipods. Additionally, a pairwise comparison was made between amphipods fed with green algae or diatoms (*Acutodesmus* sp. or *D. tenuis*). Fatty acids accounted for most (>60%) of the dissimilarities between groups. Cumulative percentage shows the percentage of variance explained by these main FA.

Comparison	Average Dis. (%)	Main FA	Cum. %
NLFA			
Wild vs. starved	37.7	16:1ω7, 18:1ω9, EPA, 16:0	69.7
Starved vs. green algae	31.0	ALA, EPA, 18:0, 18:1ω9	63.1
Starved vs. diatom	31.2	18:1ω9, 16:1ω7, EPA, 18:1ω7	59.9
Green algae vs. diatom	50.0	ALA, 18:1ω9, EPA, 18:1ω7	64.4
Green algae vs. *Acutodesmus* sp.	12.6	18:1ω9, ALA, 16:0, LA	59.9
Diatom vs. *Diatoma tenuis*	56.4	16:1ω7, 16:0, 18:1ω9	62.9
PLFA			
Wild vs. starved	14.8	DHA, 16:1ω7, 16:0, EPA	63.8
Starved vs. green algae	32.1	EPA, ALA, 18:1ω9	61.8
Starved vs. diatom	18.0	DHA, EPA, 16:0, 18:1ω7	63.6
Green algae vs. diatom	31.5	EPA, ALA, 18:1ω9, 18:1ω7	67.9
Green algae vs. *Acutodesmus* sp.	32.9	EPA, 18:1ω9, DHA, ALA	66.6
Diatom vs. *Diatoma tenuis*	49.9	16:1ω7, 16:0, 18:1ω9	70.3

**Table 3:** The contribution (±standard error) of dietary (green algae or diatom) FAs of amphipods (*P. quadrispinosa*) PLFAs and NLFAs after 7, 14, 21 and 28 days based on calculations in IsoError [55]. In cases where the contribution exceeds 100%, the amphipod δ^13^C value of FA was higher than the diet.

		SAFA			MUFA			ω-6 PUFA		ω-3 PUFA			
Treatment	Day(s)	14:0	16:0	18:0	16:1ω7	18:1ω7	18:1ω9	LA	ARA	ALA	SDA	EPA	DHA
Green, PLFA	7	51.2 ± 13.4	49.2 ± 6.5	4.6 ± 1.0	30.4 ± 3.0	82 ± 8.2	>100	>100	73.7 ± 14.6 ^1^	92.4 ± 3.3	13.6 ± 3.9	9.2 ± 7.0 ^2^	3.5 ± 1.9
	14	45.6 ± 12.2	100 ± 45	8.5 ± 1.0	65.3 ± 3.6	>100	>100	>100	>100 ^1^	98.3 ± 4.1	34.4 ± 9.2	16.6 ± 12.1 ^2^	1.8 ± 0.2
	21	76.0.0 ± 8.4	79.0 ± 7.3	8.1 ± 1.1	67.5 ± 15	>100	>100	>100	>100 ^1^	88.4 ± 10.4	19.4 ± 11.9	16.4 ± 11.6 ^2^	2.9 ± 2.6
	28	60.9 ± 19.1	80.8 ± 15.1	12.9 ± 5.4	58.2 ± 12	>100	>100	>100	>100 ^1^	>100	>100	11.2 ± 9.0 ^2^	3.6 ± 3.2
Green, NLFA	7	84.1 ± 57.9	>100	20.3 ± 4.5	86.3 ± 19.1	>100	>100	>100	73.7 ± 14.6	>100	11.6 ± 4.3	4.7 ± 15.6 ^2^	nd
	14	>100	>100	56.8 ± 21	>100	>100	>100	>100	>100 ^1^	>100	16.3 ± 10.8	76.6 ± 19.7 ^2^	nd
	21	>100	>100	35.4 ± 14.1	>100	>100	>100	>100	>100 ^1^	>100	16.1 ± 4.2	5.5 ± 15.1 ^2^	nd
	28	>100	>100	47.4 ± 17.9	>100	>100	>100	>100	>100 ^1^	>100	20.9 ± 1.6	70.1 ± 15.3 ^2^	nd
Diatom, PLFA	7	>100	35.3 ± 6.9	13.1 ± 6.2	>100	52.5 ± 9.4	64.2 ± 24.4	>100	19.6 ± 7.8 ^1^	nd	nd	73.9 ± 16.7	26.2 ± 4.0
	14	>100	40.5 ± 4.0	17.9 ± 4.0	>100	56.2 ± 8.2	93.9 ± 31.6	>100	45.2 ± 11.6 ^1^	nd	nd	>100	22.4 ± 1.5
	21	68.0 ± 0.4	27.7 ± 2.4	9.7 ± 1.3	>100	70.6 ± 8.3	>100	>100	86.1 ± 10.6 ^1^	nd	nd	>100	46.9 ± 1.7
	28	>100	58.0 ± 7.8	31.5 ± 9.1	>100	>100	>100	>100	92.2 ± 42.5 ^1^	nd	nd	>100	54.3 ± 5.5
Diatom, NLFA	7	>100	63.2 ± 10.7	8.3 ± 4.1	>100	67.2 ± 8.7	90.1 ± 33.6	90.5 ± 11.9	nd	nd	nd	>100	nd
	14	>100	63.2 ± 20.6	17.7 ± 7.3	>100	53.3 ± 8.4	>100	>100	>100 ^1^	nd	nd	>100	nd
	21	>100	24.9 ± 2.2	3,2 ± 0.5	>100	62.4 ± 7.4	49.9 ± 23.5	>100	85.5 ± 7.7 ^1^	nd	nd	>100	nd
	28	>100	69.6 ± 28	13.7 ± 6.3	>100	>100	>100	>100	nd	nd	nd	>100	nd

^1^ Calculated using δ^13^C value of dietary LA. ^2^ Calculated using δ^13^C value of dietary ALA.

## Supplementary Materials

The following are available online at https://www.mdpi.com/2218-273X/11/3/478/s1, Table S1: Phospholipid fatty acid (PLFA) profile (%) and fatty acid content (µg FA mg^−1^ DW^−1^) of *Pallaseopsis quadrispinosa* of wild or starved 12 days and fed with green algae (*Acutodesmus* sp.) or diatom (*Diatoma tenuis*) for 7, 14, 21 or 28 days. Total fatty acid (TFA) profile and content of algal diets used in the experiments. Table S2: Neutral lipid fatty acid (NLFA) profile (%) and fatty acid content (µg FA mg^−1^ DW^−1^) of P. quadrispinosa that is wild or starved 12 days and fed with green algae (*Acutodesmus* sp.) or diatom (*Diatoma tenuis*) 7, 14, 21 or 28 days. Table S3: Isotopic differences (Δ = δ^13^C_treatment_-δ^13^C_starved_) in NLFA and PLFA of amphipods.
